# Ankylosing spondylitis kyphosis surgical correction postoperative evaluation via SRS-22 domain investigation

**DOI:** 10.1186/s13018-017-0699-4

**Published:** 2018-01-09

**Authors:** Hao Zhang, XueSong Zhang, Fanqi Hu, Wenhao Hu, Yao Wang, Yongyu Hao

**Affiliations:** The Department of Orthopaedics, Chinese PLA General Hospital, Beijing, People’s Republic of China

**Keywords:** Scoliosis Research Society-22 (SRS-22), Ankylosing spondylitis kyphosis, Pedicle subtraction osteotomy, Activity, Health-related quality of life (HRQOL)

## Abstract

**Background:**

The SRS-22 is used to evaluate clinical outcomes in ankylosing spondylitis kyphosis patients. This study aimed to investigate the relationship between Scoliosis Research Society-22 (SRS-22) domains and satisfaction with management in patients who underwent surgical correction for ankylosing spondylitis kyphosis. The relationship between patient satisfaction and SRS-22 domain scores will feedback abundant information of therapeutic effect and significance for treatment guidance.

**Methods:**

In this work, 106 patients with ankylosing spondylitis kyphosis at a single institution, who underwent posterior spinal fusion of five levels or more to the sacrum, completed SRS-22 evaluation preoperatively and followed up for a minimum of 2-year postoperation. Wilcoxon tests were performed to compare preoperative with 2-year postoperative scores. Spearman correlations were investigated to evaluate associations between the 2-year treatment satisfaction and therapeutic effect in SRS-22 domain scores.

**Results:**

There were 12 females and 94 males with mean BMI of 16.4 kg/m^2^ and at the mean age of 46.3 years. All of the primary surgeries were treatments performed with mean follow-up of 26 months. A statistical improvement between paired pre- and 2-year postoperative SRS-22 domain scores and most radiographical parameters, commonly *P* ≤ 0.05, was designed and implemented. The majority of patients gave SRS-22 satisfaction score with 3.0 or more (88.5%) or 4.0 or more (68.8%), which are consistent with the moderate ceiling effect. Spearman coefficient correlations between the SRS-22 domain scores and patient satisfaction were all statistically significant, and they were from low to strong: [Mental (0.30), Activity (0.71), Pain (0.25), and Appearance (0.40)]. Furthermore, correlations for all radiographical and operative parameters were from low to strong.

**Conclusion:**

SRS-22 Activity domain correlates strongest with patient satisfaction in ankylosing spondylitis kyphosis patients who have undergone surgical correction at 2-year follow-up.

## Background

Ankylosing spondylitis (AS) is a chronic inflammatory disease, primarily involving the sacroiliac joints and spinal column, in a caudal to cranial manner. Thoracolumbar kyphosis deformity leading to cosmetic and functional impairments with a decrease in the quality of life is common in late-stage patients, resulting in the inability to look ahead, lie flat, dyspnea, and dysphagia [1]. With the progresses of the surgical technic, such as multiple Smith-Petersen osteotomy (SPO), pedicle subtraction osteotomy (PSO), vertebral column resection (VCR), and vertebral column decancellation (VCD), patients prefer operation to release from pain [2].

The Scoliosis Research Society-22 (SRS-22) questionnaire is a simple and practical disease-specific patient-based measure of treatment effectiveness for patients of spinal deformity [3–5], which has been a widely used instrument. The SRS-22 questionnaire, which measures five domains: Pain, Activity, Appearance, Mental, and Satisfaction, has already been proved to be reliable, valid, and responsive to change.

Patient satisfaction has played a vital role in the past two decades. The evolution of patient-reported outcomes (PRO) or health-related quality of life (HRQOL) outcome measures has been in parallel with an attempted change from quantity to quality of care [6]. However, there have been no previous literature expounding on the patient satisfaction with both care and outcomes after surgery for AS patients so far. The relationship between change in SRS-22 domain scores and patient satisfaction after the surgical correction of ankylosing spondylitis kyphosis has also not been reported yet. Currently, some of the orthopedic surgeons consider that the pain relief and improvement of activity function are necessary for AS kyphosis patients to increase their basic life quality. However, the others hold that the appearance correction should be paid more attention in AS kyphosis patients like that of adolescent idiopathic scoliosis (AIS) and adult deformity [7]. The target of this study is to evaluate patient satisfaction after surgical management of AS. The relationship between change in SRS-22 domain scores and patient satisfaction was investigated and analyzed in details.

## Methods

### Patients

The study was approved by the Ethics Committee of the General Hospital of People’s Liberation Army, and all patients provided informed consent. All procedures involving human participants were performed in accordance with the Declaration of Helsinki. Permissions from all the patients are received in this study. One hundred seventeen AS patients were reviewed retrospectively, from April 2011 to November 2014. The diagnosis of AS was made based on clinical features, radiographic features, and laboratory tests according to New York standards. All the patients were trapped in horizontal vision, were unable to stand upright, and suffered from chronic back pain, which seriously impacted their daily life. The primary and revision surgeries were performed for the patients. Their mean age was 46.3 (ranging from 38 to 62), and the average duration of symptoms was 23 years (ranging from 14 to 39 years). One hundred six patients (90%) of the 117 eligible patients, who were identified, completed the SRS-22 outcome instruments preoperation and 2 years postoperation.

### Planning and surgical technique

**Fig. 1:**
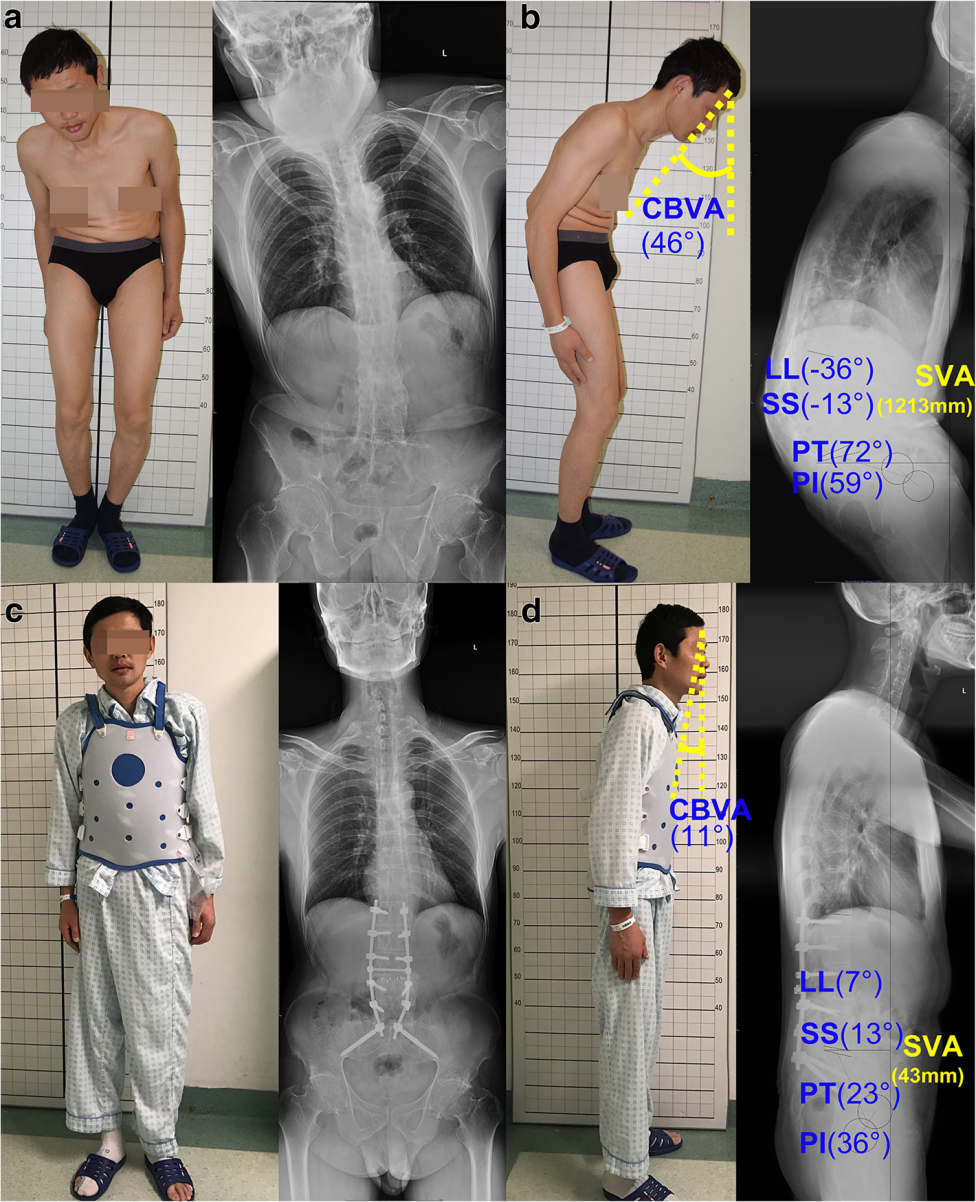
An example patient (46-year-old male) presents the severe kyphosis deformity. The most improvement of the SRS activity appears 1.5 pre-operation and 4.8 post-operation. The postoperative satisfaction with management score is 5.0. The SRS Appearance score increases from 2.0 to 4.0, the SRS pain score increases from 3.6 to 4.4, and the SRS mental health score increases from 2.8 to 4.2. Preoperative **a** posteroanterior and **b** lateral radiographs and postoperative **c** posteroanterior and **d** lateral radiographs

Single-level PSO was usually performed at L3 to regain lumbar lordosis. All the patients were operated with the same spine osteotomy (single-level PSO) (Fig. 1). According to the characteristics of the patients’ deformities, the osteotomy could be performed at L2 or L4 based on the sagittal vertical axis SVA value. The patients underwent the posterior spinal fusion of five levels or more. Regarding the osteotomy sites, 31 should be at L2, 57 at L3, and 18 at L4.

### Questionnaire method

SRS-22 is a scoliosis or spinal deformity specific questionnaire with 22 items and 5 domains: Pain, Activity, Appearance, Mental, and Satisfaction and Total Score [3, 8, 9]. Each domain score ranges from 1 to 5, with higher scores indicating better outcomes. It is the most widely used outcome instrument to measure changes in HRQOL outcomes in patients with spinal deformity and has been proved to be reliable and valid.

The SRS Satisfaction domain is calculated from two items which have five responses ranging from “Very unsatisfied” to “Very satisfied”. There are item 21 “Are you satisfied with the results of your back management?” and item 22 “Would you have the same management again if you had the same condition?” Since the SRS Satisfaction items are included in calculating the SRS Total Score, the SRS Subscore is also determined [10]. The SRS Subscore is calculated similarly without the SRS Satisfaction item inclusion. Changes in SRS domain scores are calculated by subtracting the preoperative domain scores from the 2-year domain scores.

### Radiographical data

All patients had standing anteroposterior and lateral radiographs of their whole spines which were acquired before and immediately after surgery and 2-year follow-up. The following parameters were measured on the radiographs (Fig. 1).

The radiologic measurements, containing lumbar lordosis (LL), the Cobb angle of superior endplate of L1 and inferior endplate of L5; pelvic index (PI),an anatomical parameter; pelvic tilt (PT), a pelvic positional parameter; sacral slope (SS), as the angle between the sacral plate and the horizontal line; sagittal vertical axis (SVA), as the distance between the C7 plumb line (C7PL) and the poster superior corner of S1; and the chin-brow vertical angle (CBVA), as the angle formed by the vertical line and the line drawn from the chin to the brow, were measured on the pre- and postoperative photographs of patients who could stand with the patient’s hips and knees fully extended naturally [11]. The corrections of these parameters were defined as the last follow-up measurement minus the preoperative measurement.

### Statistical analysis

Because the individual items were ordinal measures, the domain scores were treated as long ordinal measures, and therefore, a more conservative nonparametric statistical analysis was performed. Patients in a prospective database who completed the SRS-22 preoperatively and the SRS-30 1 year postoperatively were identified. Answers to the last eight questions of the SRS-30 were used as anchors to determine minimum clinically important difference (MCID) for the Pain, Appearance, and Activity domains; Subscore; and Total Score using receiver-operating-characteristic curve analysis. Calculations of MCID using distribution-based methods were also done [12]. When combined with previous reports, the results of this study in a population with ankylosing spondylitis kyphosis undergoing surgical treatment show MCID for SRS-22 scores can be estimated as 0.4. Wilcoxon tests were used to compare preoperative and 2-year postoperative domain scores. Spearman correlations were used to evaluate associations between the 2-year postoperative SRS-22 Satisfaction score and changes in the SRS-22 Total Score, Subscore, Pain, Activity, Appearance, and Mental domain scores; and all radiographic parameters from pre- to 2-year postoperation (Table 1). Correlation coefficients (*r*) ranging from 0 to 0.19 were considered very weak, 0.20 to 0.39 as weak, 0.40 to 0.59 as moderate, 0.60 to 0.79 as strong, and 0.80 to 1 as very strong. And we also made a heat map of the SRS-22 domain scores and radiographical parameters to clarify correlations more clearly (Fig. 4).

**Table 1:** Spearman coefficient correlations between the change in SRS-22 domain scores or radiographical parameters and SRS-22 Satisfaction score at 2-year postoperative

Parameters	*r*	*P*
Follow-up	− 0.13	0.19
Diagnosis	− 0.02	0.74
Number of comorbidities	0.06	0.55
BMI	0.01	0.92
Smoking	0.07	0.419
Osteotomies	− 0.04	0.635
Level of surgery	0.10	0.24
Operative time	0.08	0.38
SRS-22 Pain change	0.25	0.03
SRS-22 Appearance change	0.40	0.00
SRS-22 Activity change	0.71	0.00
SRS-22 Mental change	0.30	0.01
SRS-22 Subscore	0.35	0.00
Radiographical parameters		
CBVA change	− 0.44	0.00
SVA change	− 0.60	0.00
LL change	0.61	0.00
SS change	0.42	0.00
PT change	− 0.30	0.01
PI change	− 0.27	0.02
PI − LL change	− 0.44	0.00

*P* values < 0.05 were considered statistically significant for all comparisons. Spearman coefficient (*r*) correlations ranging from 0 to 0.19 were considered very weak, 0.20 to 0.39 as weak, 0.40 to 0.59 as moderate, 0.60 to 0.79 as strong, and 0.80 to 1 as very strong

All statistical analyses were done by using SPSS (version 17.0, SPSS Inc., Chicago, IL).

## Results

**Fig. 2:**
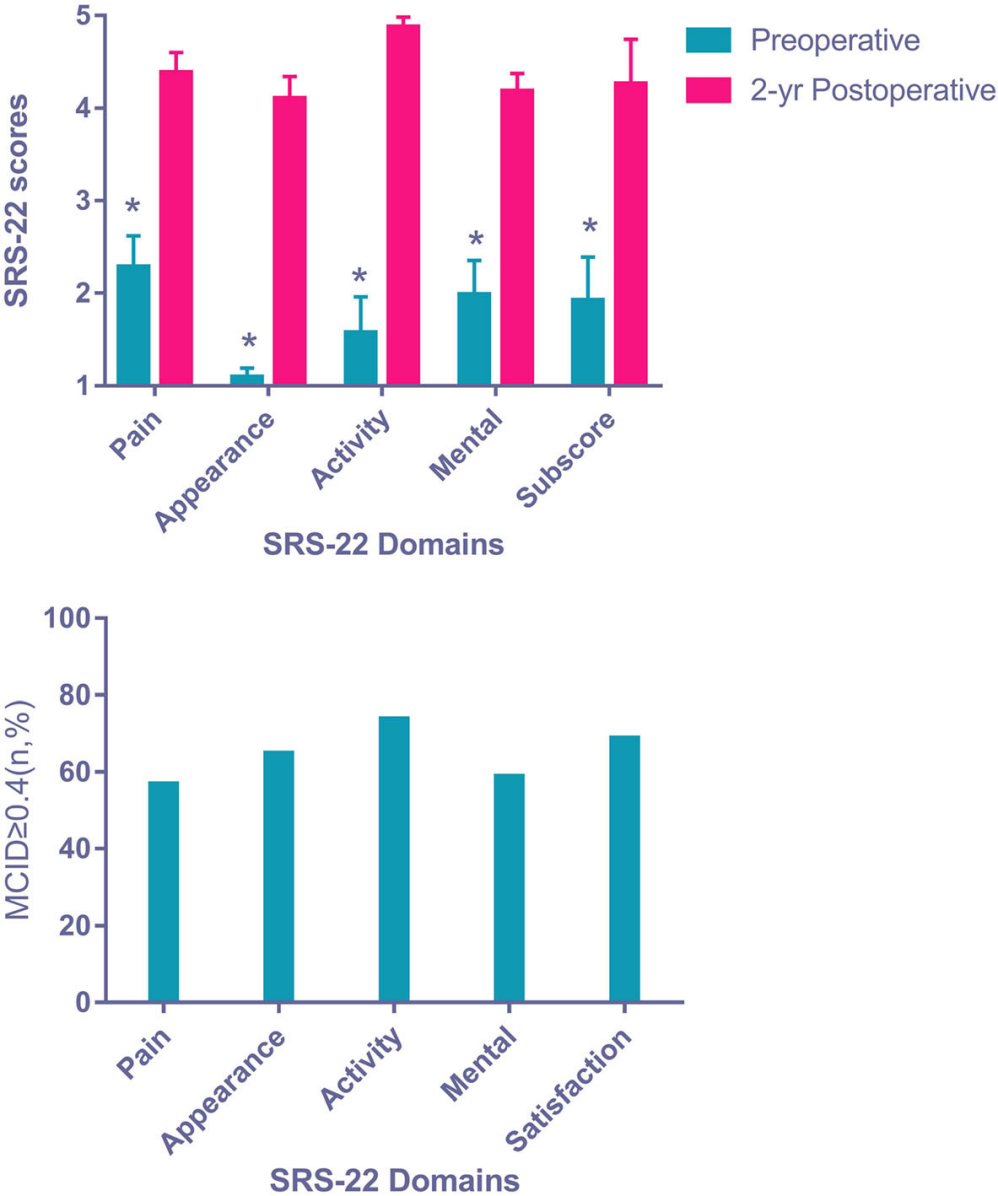
Comparison of scores of SRS-22 before and 2 years after surgery and number of patients achieving MCID thresholds for each domain (*n* = 106).*P values < 0.05 were considered statistically significant for all comparisons

There were 12 females and 94 males with a mean BMI of 16.4 kg/m^2^ and mean age of 46.3 years. There were all primary surgeries with mean follow-up of 26 months. The operating time was 232 ± 52 min for spine osteotomy surgery. The blood loss was 1240 ± 542 ml. All osteotomies were performed in the lower thoracic and lumbar segments (T12–L3). All patients could walk with horizontal vision and lie on their backs postoperatively. Using a minimum clinically important difference (MCID) threshold for the pre- to postoperative change for each domain score of 0.4, the percentage of patients reaching MCID was calculated (Fig. 2). The proportion of patients reaching MCID was highest for SRS-22 Activity (73%).

**Fig. 3:**
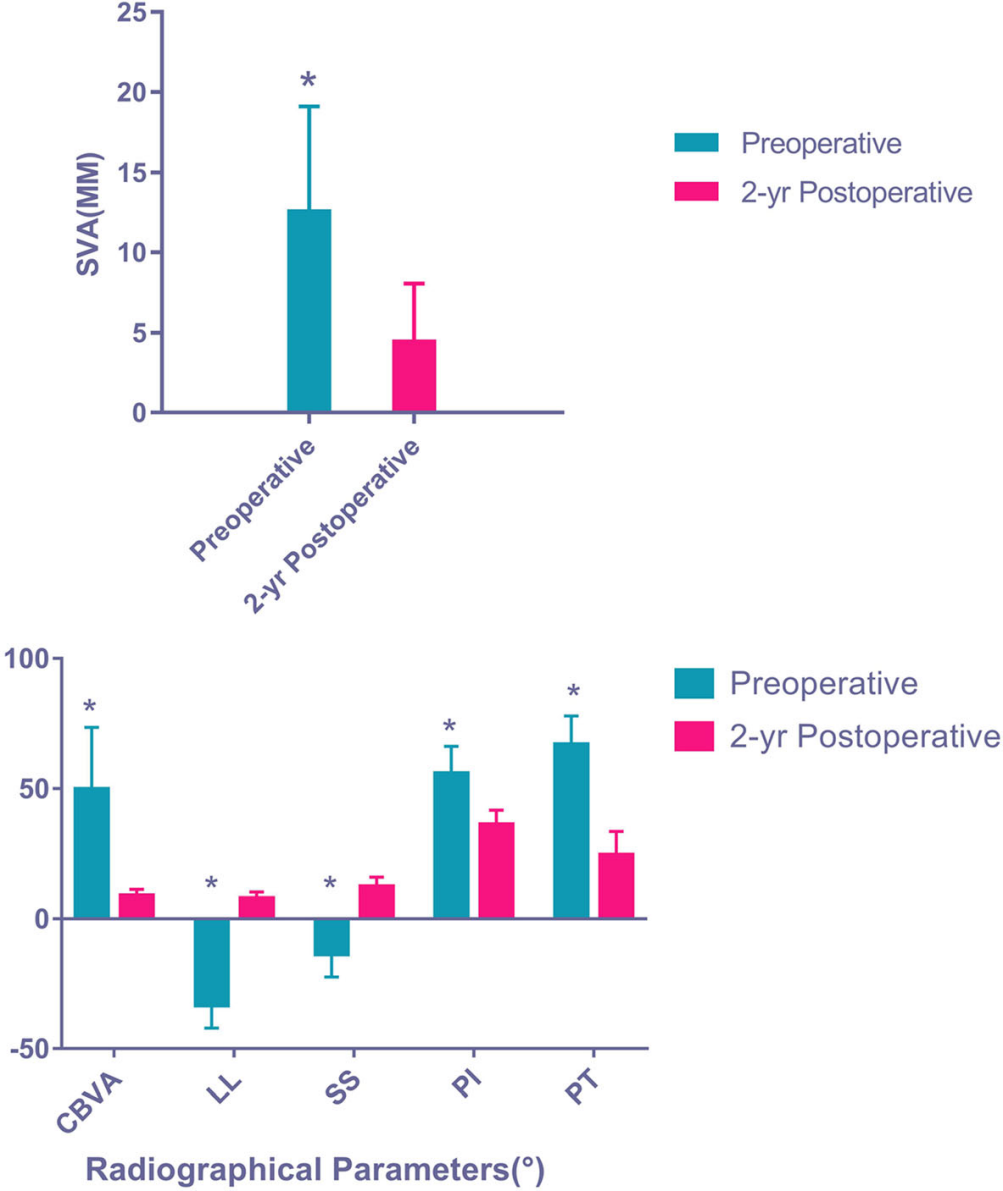
Comparison of mean radiographical measures preoperatively and 2 years postoperatively. All comparisons were statistically significant (*P < 0.05)

There was a statistically significant change in most of the radiographical parameters (Fig. 3). Additionally, there was a statistically significant improvement between all paired pre- and 2-year postoperative SRS-22 domain scores [Mental (0.30), Activity (0.71), Pain (0.25), moderate (Appearance (0.40), SRS-22R Subscore (0.35))], with the greatest improvement in the SRS-22R Activity domain (*P* < 0.05). The majority of patients had a 2-year Satisfaction score 4.0 or more (68.8%) or 3.0 or more (88.5%) (Table 1). Spearman correlations between the change in domain scores or radiographical parameters and the SRS-22 Satisfaction score at 2 years postoperative were calculated for 20 different parameters. Twelve of the 20 parameters (60%) analyzed were statistically significant with correlation coefficients ranging from 0.01 and 0.66, indicating weak to strong correlations (Table 1).Correlation between the 2-year change in SRS-22 Activity domain and the 2-year postoperative SRS-22 Satisfaction score (0.71) was the strongest. The next greatest correlation was between the 2-year change in SRS Appearance score and the 2-year postoperative SRS Satisfaction score (0.40); SRS-22 Mental score (0.30) and Pain score (0.25) had a weak correlation. The rest were either weak or very weak. The postoperative radiographical data of the patients were significantly improved, and the degree of improvement was also significantly. The strongest correlation was the SVA change (− 0.60) and the LL change (0.61). The CBVA change (− 0.44), the SS change (0.38) and the PI − LL change (− 0.44) had a moderate correlation. And the PT change (− 0.30) and the PI change (− 0.31) had a weak correlation. (Table 1).

## Discussion

Nowadays, there is a growing concern over the patient satisfaction in spine surgery. More and more medical units consider patient satisfaction to evaluate the quality of medical services. So, the normative assessment of factors affecting the performance of AS patients is significant to promote medical services with humanism [6]. In the Chinese population of patients with AS undergoing surgical correction, there are critical correlations between the change in the SRS-22 Satisfaction score and the SRS-22 four domain scores, similar to the change in the SRS-22 Satisfaction score and radiographical parameters. In Asher’s original study on the SRS-22 including 58 patients with scoliosis, the author reported a correlation coefficient of 0.66 between the Satisfaction and Appearance domains and a correlation coefficient of 0.67 between Satisfaction and SRS-22R Subscore which indicates strong correlations [3, 4]. Likewise, in a multicenter study of 745 patients with AIS, Carreon et al. found that Subscore and Appearance had the strongest correlation in the study investigating patient satisfaction after surgical correction of AIS [10]. Similar to the adolescent population, in a study of 135 patients with adult spine deformity, the author found that SRS-22R appearance correlated most with patient satisfaction in adult deformity patients undergoing five or more level fusions to the sacrum at 5-year follow-up [7].

**Fig. 4:**
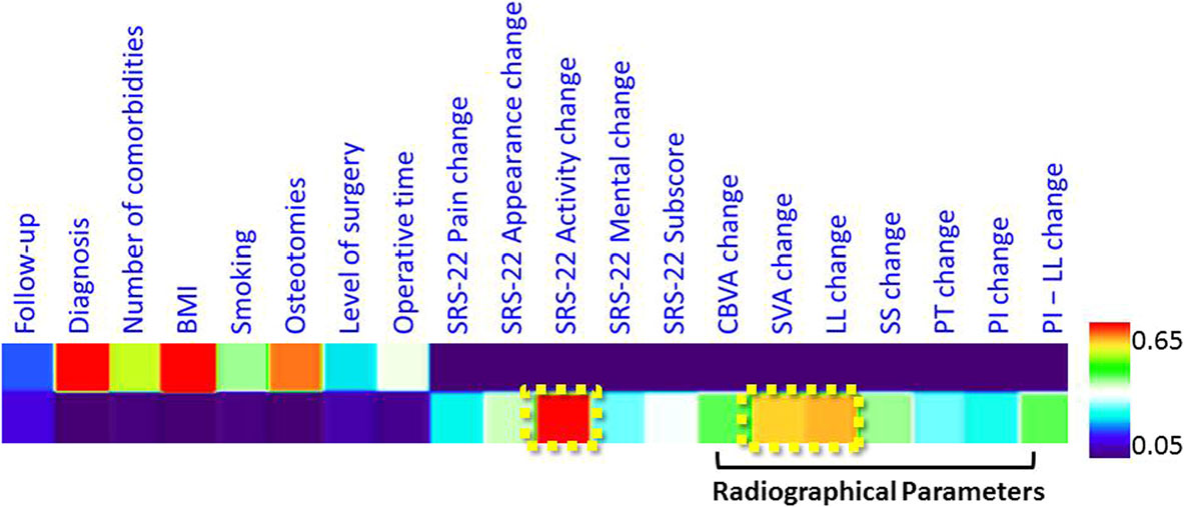
Heat map of the SRS-22 domain scores and radiographical parameters. It shows the evolution of the signals (quenching/enhancing), suggesting high variance in the response

AS osteotomy surgery, as well as adolescent idiopathic scoliosis and adult spine deformity surgery, were adopted to remedy the appearance problems in most cases. In contrast, in our study, SRS Activity domain was obviously observed among all the SRS domains in AS patient groups. Our study calculated the percentage of patients reaching MCID for each domain (Fig. 2), and Appearance, Pain, and Mental domains had a lower proportion of patients reaching MCID by using an MCID threshold of 0.5 for all domains. The weak to strong correlations observed between the change in SRS-22 domain scores and the 2-year SRS-22 Satisfaction score may also be attributable to the prominent ceiling effect in the Satisfaction domain. Although no floor effects were observed, a significant ceiling effect for the Satisfaction domain was seen in the current study, with 33% of patients scoring a 5 and 68.8% scoring greater than 4 (Table 2) [9]. The weak to moderate correlations may also be due to the lack of responsiveness of the SRS-22 to measure clinically relevant changes in pain and mental health 2 years after correction of kyphosis in the AS population. Although the changes in domain scores were statistically significant, the mathematical values of the change in scores were small and similar to the sample population from which the SRS-22 was initially studied. It may actually have been significant clinically relevant change in these domains after orthomorphia when the changes in Appearance, Pain and Mental health truly responded in SRS-22. The data from studies examining the relationship between radiographic parameters and satisfaction outcomes demonstrate a considerably strong, linear correlation between satisfaction and SVA change and LL change. This phenomenon reminds us that the orthopedist may ignore the patient’s postoperative functional recovery and the quality of life when they excessively pursue the deformity correction and the imaging improvements during the operation. And longer fusion segments can indeed better correct the deformity, but the impact on the motion of spinal surgery patients should not be ignored. In our opinion, the change of the SVA and the LL need more attention in the course of surgery (Fig. 4). Global spinal realignment should attempt to obtain postoperative SVA < 50 mm and LL = PI± 9° [13], and we require ambition to balance radiographic parameters with good clinical judgment, so that we can gain more patient satisfaction and postoperative outcomes.

**Table 2:** Distribution of SRS-22R Satisfaction score

Satisfaction score	Frequency	Percentage	Cumulative percentage
5.0	35	33.0	33
4.5	17	16.0	48
4.0	22	20.8	68.8
3.5	8	7.5	76.3
3.0	13	12.2	88.5
2.5	3	2.8	91.3
2.0	6	5.7	97
1.5	1	0.9	97.9
1.0	1	0.9	100

In the AS population, the surgical correction of their kyphosis may have a limited impact on their activity, self-image, mental health, and satisfaction when compared with the larger role that educational, physiological, socio-cultural, and biopsychological factors play. In our conversation with patients, the improvement of the patient’s activities by operation is much greater than that of the appearance. But it does not reflect a statistically significant difference in mathematics. Therefore, this study offers a fundamental principle to improve the AS deficiencies via SRS-22 investigation. What is more, a number of other factors also affect the relatives’ satisfaction with the degree of overall treatment outcomes, such as experience of patients and relatives during the hospitalization, relationships with the surgeon and the surgeon’s assistants, and so on. The patients’ case information will feedback more advantages.

## Conclusions

In summary, the scale of the evaluation of SRS-22 Activity domain can present the treatment of patient satisfaction. Harmony among spinopelvic parameters is one of the important factors. The activity of patients with kyphosis in ankylosing spondylitis is one of the key factors to evaluate a successful treatment. Different with traditional awareness, appearance is supposed to be the most critical consideration to plan a surgical strategy. This work can guide clinical spine surgeons with patients’ satisfaction expectations and balance all the surgical correction parameters.

## Abbreviations

AS: Ankylosing spondylitis
C7PL: C7 plumb line
CBVA: Chin-brow vertical angle
HRQOL: Health-related quality of life
LL: Lumbar lordosis
PI: Pelvic index
PSO: Pedicle subtraction osteotomy
PT: Pelvic tilt
SRS-22: Scoliosis Research Society-22
SVA: Sagittal vertical axis
VCD: Vertebral column decancellation
